# Barriers and facilitators to implementation of epilepsy self-management programs: a systematic review using qualitative evidence synthesis methods

**DOI:** 10.1186/s13643-020-01322-9

**Published:** 2020-04-25

**Authors:** Allison A. Lewinski, Abigail Shapiro, Jennifer M. Gierisch, Karen M. Goldstein, Dan V. Blalock, Matthew W. Luedke, Adelaide M. Gordon, Hayden B. Bosworth, Connor Drake, Jeffrey D. Lewis, Saurabh R. Sinha, Aatif M. Husain, Tung T. Tran, Megan G. Van Noord, John W. Williams

**Affiliations:** 1 Durham Center of Innovation to Accelerate Discovery and Practice Transformation, Durham Veterans Affairs Medical Center, Durham, NC USA; 2 Cooperative Studies Program Epidemiology Center-Durham, Durham Veterans Affairs Medical Center, Durham, NC USA; 3 Department of Population Health Sciences, Duke University School of Medicine, Durham, NC USA; 4 Division of General Internal Medicine, Department of Medicine, Duke University School of Medicine, Durham, NC USA; 5 Department of Psychiatry and Behavioral Sciences, Duke University School of Medicine, Durham, NC USA; 6 Department of Neurology, Duke University Medical Center, Durham, NC USA; 7 Neurodiagonostic Center, Durham Veterans Affairs Medical Center, Durham, NC USA; 8 School of Nursing, Duke University, Durham, NC USA; 9 Department of Health Policy and Management, Gillings School of Global Public Health, University of North Carolina at Chapel Hill, Chapel Hill, NC USA; 10 Center for Personalized Health Care, Duke University School of Medicine, Durham, NC USA; 11 Department of Neurology, Uniformed Services University School of Medicine, Bethesda, MD USA; 12 Neuroscience Medicine, Duke Clinical Research Institute, Duke University, Durham, NC USA; 13 University of California at Davis, Davis, CA USA

**Keywords:** Epilepsy, Self-management, Qualitative research

## Abstract

**Background:**

Epilepsy affects nearly 50 million people worldwide. Self-management is critical for individuals with epilepsy in order to maintain optimal physical, cognitive, and emotional health. Implementing and adopting a self-management program requires considering many factors at the person, program, and systems levels. We conducted a systematic review of qualitative and mixed-methods studies to identify facilitators and barriers that impact implementation and adoption of self-management programs for adults with epilepsy.

**Methods:**

We used established systematic review methodologies for qualitative and mixed-methods studies. We included studies addressing facilitators (i.e., factors that aided) or barriers (i.e., factors that impeded) to implementation and adoption of self-management interventions for adults with epilepsy. We conducted a narrative thematic synthesis to identify facilitators and barriers.

**Results:**

The literature search identified 2700 citations; 13 studies met eligibility criteria. Our synthesis identified five themes that categorize facilitators and barriers to successful implementation epilepsy self-management: (1) *relevance*, intervention content that facilitates acquisition of self-management skills; (2) *personalization*, intervention components that account for the individual’s social, physical, and environmental characteristics; (3) *intervention components*, components and dosing of the intervention; (4) *technology considerations*, considerations that account for individual’s use, familiarity with, and ownership of technology; and (5) *clinician interventionist*, role and preparation of the individual who leads intervention. We identified facilitators in 11 of the 13 studies and barriers in 11 of the 13 studies and classified these by social-ecological level (i.e., patient/caregiver, program, site/system).

**Conclusion:**

Identification of facilitators and barriers at multiple levels provides insight into disease-specific factors that influence implementation and adoption of self-management programs for individuals with epilepsy. Our findings indicate that involving individuals with epilepsy and their caregivers in intervention development, and then tailoring intervention content during the intervention, can help ensure the content is relevant to intervention participants. Our findings also indicate the role of the clinician (i.e., the individual who provides self-management education) is important to intervention implementation, and key issues with clinicians were identified as barriers and opportunities for improvement. Overall, our findings have practical value for those seeking to implement and adopt self-management interventions for epilepsy and other chronic illnesses.

**Systematic review registration:**

PROSPERO registration number is CRD42018098604.

Contributions to the literature

- Patient, caregiver, and clinician involvement may improve implementation and adoption of epilepsy self-management interventions
- A flexible self-management approach with tailoring to individual patients may address concerns regarding cognitive limitations, use of technology, and relevance of self-management information
- Clinicians involved in self-management interventions need appropriate training, dedicated clinical time, and to be provided with clearly written standardized protocols that articulate the clinician’s role in the intervention
- Technology use for self-management is highly dependent on individual characteristics such as familiarity and ownership of technological devices (e.g., mobile phones, computers)

## Background

The World Health Organization estimates that epilepsy affects about 50 million people worldwide [1]. Individuals with epilepsy have poorer health outcomes and report a poorer quality of life [2–4]. Self-management is important for individuals with epilepsy in order to maintain optimal physical, cognitive, and emotional health following diagnosis [5, 6]. Engagement in self-management may increase self-efficacy, medication adherence, avoidance of seizure triggers, and improves patient and family knowledge about when to seek urgent medical care [7, 8]. For instance, education based-approaches positively impact self-management, whereas psychosocial therapy-based approach positively impact quality of life [9, 10]. A recent Cochrane review identified weak support for intervention strategies such as using epilepsy-trained nurses and educational interventions to support self-management in adults with epilepsy [11]. Yet, rates of self-management and adherence to treatment regimens remain suboptimal among individuals with epilepsy, leading to increased medical care and premature death [5, 6].

As with many chronic health conditions, challenges to self-management for adults with epilepsy include low health literacy, poor social support, low levels of education, medication and disease-related side effects, and low socioeconomic status [12–18]. Patients with epilepsy have additional disease-specific self-management concerns which can impact engagement in, and adherence to, self-management [19]. The effectiveness of self-management in adults with epilepsy may be influenced by commonly co-occurring medical conditions such as traumatic brain injury, mood disorders, associated cognitive impairment, and/or impulse-control issues. Further complicating self-management plans, high levels of disease-related stigma, driving or mobility restrictions, and social isolation may serve as barriers to attending intensive epilepsy self-management programs and/or medical appointments that occur in-person and/or require a sustained time commitment [20–22]. Thus, engagement in self-management and treatment adherence may be positively influenced when self-management programs are informed by knowledge of epilepsy-specific considerations and how these considerations may influence self-management.

A possible solution to epilepsy-specific self-management concerns may be self-management programs offered within the context of clinicians providing health care for patients with epilepsy. Implementing self-management programs in health care systems requires consideration of many multi-level factors [23]. Facilitators and barriers to implementation can occur at any level of an intervention, including the person (patient or caregiver), program, and system levels. Research has shown the importance of allocating resources, balancing context-specific adaptation and program fidelity, and involving key stakeholders during initial and sustained implementation and adoption [24–27]. Although scientific organizations recommend self-management support [28–30], there is little guidance about how to implement these programs, and we identified no prior systematic reviews that focused on this issue.

We conducted a systematic review of quantitative, qualitative, and mixed-methods studies to examine the barriers and facilitators to implementing and adopting epilepsy self-management programs in large health care systems. We chose qualitative evidence synthesis methods to incorporate evidence from multiple study designs that addressed the same overarching concept of implementation and adoption of epilepsy self-management programs. Our study addresses gaps in understanding facilitators and barriers at the person, program, and system levels that impact implementation and adoption of epilepsy self-management programs.

## Methods

This systematic review was part of a Veterans Health Administration (VHA)–funded report available online (www.hsrd.research.va.gov/publications/esp) [9, 10]. The purpose of the systematic review was to address gaps in evidence regarding the efficacy and effectiveness of self-management programs for adults with epilepsy, synthesize the current evidence on self-management programs for patients with epilepsy, and identify potential barriers in the adoption of these programs within a health care system. We developed and followed a standard protocol for this review in collaboration with stakeholders (PROSPERO: CRD42018098604). We adhered to the Preferred Reporting Items for Systematic Reviews and Meta Analyses (PRISMA) guidelines [31] (Additional file 1).

### Literature search and study selection

The conceptual model, literature search, and study selection methods for the systematic review are described in greater detail in the full monograph [9, 10]. A full list of inclusion and exclusion criteria (i.e., population, intervention, comparator, outcome, timing, and setting) is listed in Table 1. The major criteria for eligibility included randomized or quasi-experimental studies that enrolled adults with epilepsy and evaluated self-management interventions and reported a relevant clinical, process, or economic outcome. Eligible interventions were required to have a primary focus on self-management of epilepsy, could not be education-only or general medical care interventions, and could not focus primarily on a comorbid psychological diagnosis. Additionally, eligible studies focused on adults (aged ≥ 18) with new or chronic epilepsy, family members and/or caregivers of adults with epilepsy, and/or stakeholders involved in implementation of epilepsy self-management programs. For this specific analysis, we included additional observational designs and qualitative studies that addressed facilitators and barriers to intervention implementation and adoption. We operationalized self-management as programs that facilitated the provision of epilepsy-specific knowledge and skills that assisted an individual and/or caregiver in incorporating epilepsy self-management behaviors into daily life. Our definition was based upon a conceptualization of self-management by Jonkman and colleagues [32]; however, we modified the definition to only include one component beyond knowledge acquisition [9, 10]. We operationalized implementation and adoption as the uptake, and continued use, of an evidence-based intervention in health care practice [34].

**Table 1:** Eligibility criteria (reproduced with permission from Luedke et al. [9])

Study Characteristic	Include	Exclude
Population	• Adults (aged ≥ 18) with new or chronic epilepsy • Family members and/or caregivers of those with epilepsy • Stakeholders involved in implementation (e.g., neurologists, health coaches, nurses, administrators)	• Children • Populations with < 70% adults • Severe learning disabilities • Non-epileptic seizures (i.e., psychogenic seizures) • Populations who have been recruited for depression or who have major mental illness (e.g., bipolar, major depressive disorder, schizophrenia)
Intervention	Self-management defined as interventions that aim to equip patients with skills to actively participate and take responsibility in the management of epilepsy in order to function optimally through at least knowledge acquisition and a combination of 1 or more of the following: • Stimulation of independent sign/symptom monitoring • Medication management • Enhancing problem-solving and decision-making skills for epilepsy treatment management, safety promotion (e.g., driving) • Changing health behaviors (including stress management, sleep, substance use)^a^ Examples include: • Psychoeducation (e.g., cognitive behavioral therapy) • Behavioral interventions (e.g., adherence strategy training) • Personalized care plan development and coaching	• Multicomponent interventions that include self-management but where self-management is not the primary intervention • Cognitive behavioral therapy focused on comorbid mental illness in patients with epilepsy (e.g., depression in patients with epilepsy) • Education-only interventions • General care delivery interventions (e.g., introducing specialist nurse practitioner or implementation of clinical practice guidelines)
Comparator	Any (usual care, attention control, active intervention)	None
Outcomes	Any relevant clinical, process, or economic outcome to epilepsy self-management interventions	None
Timing	Any	Any
Setting	• Delivered in person (individual or group) in outpatient settings, or remotely via telehealth technology (e.g., mobile or internet) • Delivered by health care team members or trained lay workers	• Inpatient • Delivered only in emergency departments
Design^b^	• Randomized trials • Nonrandomized trials • Controlled before-after studies^b^ • Prospective cohort study if it includes a properly adjusted analysis • Qualitative and survey designs if specifically addressing facilitators and barriers to adoption of epilepsy self-management interventions	• Self-described pilot studies and/or sample size < 20 • Studies with retrospective data collection • Interrupted time series • Case series • Systematic reviews/meta-analyses • Reports that do not include primary data on barriers or facilitators
Language	English	Non-English
Countries	OECD^c^	Non-OECD
Years	Any	None
Publication types	Full publication in a peer-reviewed journal	Letters, editorials, reviews, dissertations, meeting abstracts, protocols without results

^a^Adapted from Jonkman et al. [32]

^b^See Cochrane EPOC criteria for definitions and details [33]

^c^*OECD* Organization for Economic Cooperation and Development includes Australia, Austria, Belgium, Canada, Chile, Czech Republic, Denmark, Estonia, Finland, France, Germany, Greece, Hungary, Iceland, Ireland, Israel, Italy, Japan, Korea, Latvia, Luxembourg, Mexico, Netherlands, New Zealand, Norway, Poland, Portugal, Slovak Republic, Slovenia, Spain, Sweden, Switzerland, Turkey, United Kingdom, United States

For this systematic review, we collaborated with a medical librarian to develop a search strategy for each database (Additional file 2) [9, 10]. We searched MEDLINE® (via PubMed®), PsycINFO, Cochrane Central Register of Controlled Trials (CENTRAL), and CINAHL in April 2018, and the MEDLINE search was updated in March 2019. Citations and the full-text of potentially eligible studies were evaluated by two investigators. We resolved disagreements using consensus between two investigators, and involved a third investigator as needed. All articles that met eligibility requirements and addressed facilitators and barriers to implementation and adoption were included for data abstraction.

### Data extraction strategy

Data abstraction was completed by a team of two co-investigators (AAL, AS) who had experience in qualitative methodology, under the guidance of the primary investigator (JWW). Data from published reports were entered into a Microsoft Excel spreadsheet created for this project. Abstracted data included study purpose, study design and details, participant eligibility criteria, recruitment strategy, control group, participant type (i.e., patient, caregiver, clinician, researcher), sample size and demographics, main findings salient to facilitators and barriers of implementation and adoption, and other comments. We abstracted implementation and adoption barriers (i.e., description of themes or factors that impeded the use and implementation of the intervention as reported in the study’s results and/or findings sections) and facilitators (i.e., description of themes or factors that aided the use of the intervention as reported in the study’s results and/or findings sections) to the implementation of self-management interventions (as distinct from barriers and facilitators of an individual engaging in self-management behaviors). Data were entered independently by one investigator, and then independently reviewed by a second investigator. Following the completion of data entry, the two investigators met to discuss abstracted data; disagreements were resolved by consensus.

#### Risk of bias

To evaluate the risk of bias (ROB) across included studies, we employed ROB instruments appropriate for each study design (e.g., qualitative, cross-sectional, mixed-methods) [35, 36] (Additional file 3). The evaluation of ROB in each study was based on an assessment of whether the study was well-designed and conducted within the research paradigm proposed by the study authors. These tools assess risk of bias in 5 to 10 domains, categorizing each domain as low, unclear, or high ROB. The tools are not intended to create summative scores [35, 36], and so we applied a more holistic approach by considering the individual domains and overall impressions of the study. First, each study was independently rated by each qualitative investigator (AAL, AS) using a ROB tool. Then, using this first ROB rating, the two investigators met and discussed each study in the context of valid and rigorous qualitative research methodology [37–39]. For instance, we discussed *sampling technique* (e.g., did the authors describe recruitment and sample selection in detail? Did the sampling align with the population described in the introduction?); *analysis* (e.g., Was analysis completed by one individual or more than one individual? Was analysis completed by the individual who completed data collection?); and *measures* (e.g., Does the data collection method align with the introduction and purpose of the study?). After discussing each study, we assigned an overall ROB using the following definitions: (1) *low ROB*—bias, if present, is unlikely to alter the results seriously; (2) *unclear ROB*—a risk of bias that raises some doubts about the results; and (3) *high ROB*—bias may alter the results seriously. Disagreements were resolved by consensus between the two qualitative investigators and the senior author. We did not validate each ROB tool for use in our systematic review as we did not make any real change to the intent or the goal of each instrument.

We used the 10-item Critical Appraisal Skills Programme (CASP) for Qualitative Research Studies [35] for the included qualitative studies (*n* = 5). Each item is rated “Yes,” “No,” or “Can’t tell”; there is no summary rating. The overall ROB in the 5 qualitative studies was low [22, 40–43]. For the quantitative descriptive studies, we used the 5 items in the Mixed Methods Appraisal Tool (MMAT) [36]. These criteria address the sampling strategy, the sample representativeness, measurements, risk of nonresponse bias, and appropriateness of the statistical analysis. The MMAT rates each item “Yes,” “No,” or “Can’t tell”; there is no summary rating. We used the 5-item MMAT specific to mixed-methods studies [36] for the mixed-methods study (*n* = 1). These criteria address the rationale for using mixed-methods, the integration of the study components, the interpretation of the study components, discussion of divergences or inconsistencies between the quantitative and qualitative data, and how each component of the study adheres to the quality criteria of each of the quantitative and qualitative methods. Similar to the quantitative descriptive studies, the MMAT for mixed-methods studies rates each item “Yes,” “No,” or “Can’t tell”; there is no summary rating.

### Data synthesis and presentation

Data synthesis was completed by the same co-investigators under the guidance of the primary investigator. We analyzed the abstracted data using thematic synthesis and the framework method [44, 45]. Thematic synthesis utilizes a three-stage method for data synthesis: (1) free coding of findings in the included studies, (2) organizing data into descriptive themes, and (3) generating analytical themes that combine the findings of individual studies into interpretations that cross the findings of the studies [45]. The framework method is a complementary approach to management and analysis of qualitative data, which relies on charting the data into a matrix based on descriptive and analytic codes [44]. Using the research question and our full-text review and familiarization with these data as a guide, we created a framework based on three levels from the social ecological framework [46] that included barriers and facilitators as reported for a category (i.e., patient with epilepsy or caregiver, program or intervention, and site or health system). Then, we described each piece of data either as being a facilitator or a barrier to implementation of a self-management program. Each piece of data was subsequently grouped into an ecological framework level (i.e., patient/caregiver, program/intervention, site/health system). We then completed first-level analysis of these data and confirmed the validity of our interpretations by referencing the original texts. After the data were independently coded and discussed among the two qualitative researchers, we conducted thematic synthesis by identifying and grouping related codes within each category (e.g., patient/caregiver, program/intervention, site/health system)*.* Then, each researcher independently organized related codes into themes. We reviewed the themes and then identified overarching themes that applied to both facilitators and barriers. The creation and identification of codes and themes was iterative. To ensure rigor and validity of these findings, we independently coded and developed themes. We then discussed theme development and identification until we reached agreement between the two researchers. Next, we conducted a narrative synthesis of the data and displayed its summary in a crosstab of themes by study.

#### Ecological levels

For each facilitator and barrier, we first identified the respondent (e.g., the patient with epilepsy, caregiver, or health care clinician) associated with each theme. Then we examined each theme at one of three levels, adapted from ecological models of health behavior. The ecological models of health emphasize that determinants of behavior derive from individuals and characteristics of their environments that influence behavior directly and in interaction with one another [46]. Table 2 provides operationalized definitions and exemplars for each of the three ecological levels. These categories were mutually exclusive; thus, each facilitator and barrier could only be placed in one category (i.e., patient/caregiver, program/intervention, site/health system).

**Table 2:** Definitions and examples of the ecological levels

Level	Definition	Examples
Person	The individual characteristics of the patient or caregiver who is engaging in the epilepsy self-management intervention	• Individual’s current engagement in self-management behaviors [47] • Ability of the individual to obtain self-management skills [40]
Program	Self-management intervention being implemented and evaluated	• Provision of relevant topics that enabled self-management skill acquisition [42, 48] • Involving caregivers in the intervention [49]
Site/system	Health care site or system where the self-management intervention is being implemented and evaluated that highlights the usability of the intervention	• Uniform program standards [42] • Provider characteristics such as job role within the organization [50]

## Results

**Fig. 1:**
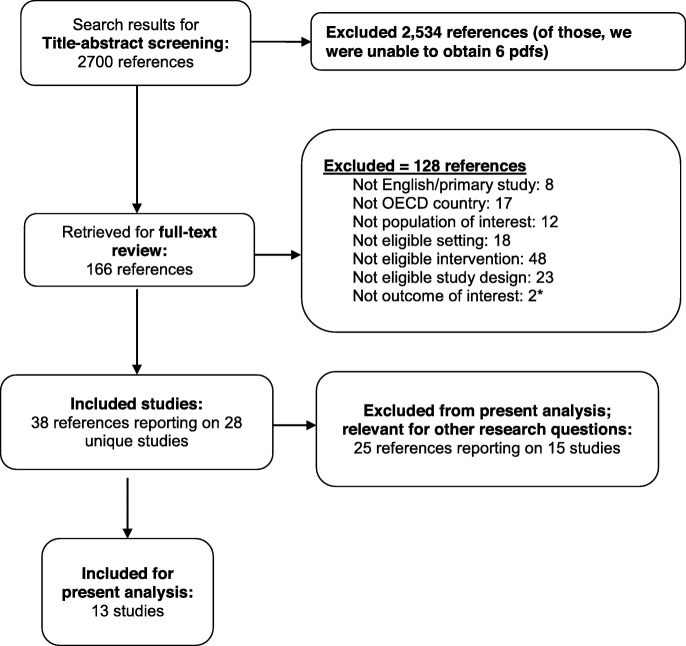
PRISMA literature. *OECD*, Organization for Economic Cooperation and Development. Asterisk means reference did not report a relevant clinical, process, or economic outcome

We identified 13 studies addressing facilitators and barriers to implementing self-management interventions for persons with epilepsy (Fig. 1) [22, 40–43, 47–54]. Study designs in this analysis included semi-structured interview (*n* = 5) [22, 40–43]; cross-sectional survey (*n* = 5) [47, 48, 50, 53, 54]; longitudinal survey (*n* = 1) [52], mixed-methods including group semi-structured interview, cross-sectional survey, and records review (*n* = 1) [49], and a discrete choice experiment (*n* = 1) [51]. Study respondents included patients with epilepsy, caregivers, and health care clinicians together [42, 49, 52–54], patients and caregivers only [22, 40, 41, 43, 47, 48, 51], and health care clinicians [50]. We describe study characteristics including study sample, epilepsy type, and intervention in Table 3.

**Table 3:** Study characteristics

Study # Enrolled Study design (companion)	Design details	Eligibility Recruitment detail	Age: mean (SD) Female: number (%)	Finding(s)	Risk of bias
Descriptive quantitative studies					
Atkinson-Clark [51] 299 Discrete-choice experiment	Repetitive choice between 2 hypothetical SM programs, varying on 6 characteristics	Adults 18 + self-report diagnosed with epilepsy 100 adults targeted in each of: France, Netherlands, Germany. Methods of recruitment NR.	Age: 45.5 (17.6), Female: 161 (53.8)	• Out of pocket cost to participant is the most important feature for participation, (*p* < .01) • Preferences vary by categorical groups, including by disease burden (high/low), country (France vs. Germany vs. Netherlands), and SES • 20% of participants preferred to never participate in SM programs	*Sample Strategy*: low *Representative sample*: unclear *Appropriate measures*: low *Nonresponse bias*: low *Appropriate analysis*: low *Summary*: unclear how well panels represented the population of interest
Begley [52] Single group longitudinal study of two successive clinic visits; process measures included exit interviews; quantitative questionnaire used in at 2 time points Begley [54]	Feasibility assessed by giving surveys to patients and providers after visits to obtain ease of use and usefulness of MINDSET, quality and concurrence of patient/provider communication/shared decision making, and capacity of tool to monitor epilepsy characteristics and self-management behaviors over time	Diagnosis of epilepsy, aged 18 or over, spoke English Recruited from 3 epilepsy clinics; patients identified by collaborating providers	Age: 36.9 (10.9) Female: 14 (66.7%)	• Majority of patients noted time to use MINDSET was “just right” and found MINDSET to be more understandable over time; majority of patients needed assistance to use MINDSET the first time, and the duration for patients to enter data into MINDSET was between 11 min to 1 h • Process: Incident log noted 9 occurrences of technical/user problems in visit 1 • Exit interviews: Patients positive about MINDSET; improvements include clarity and wording, applicability of behavioral items, accounting for age differences; patients found action plan more helpful after visit 2 action plan printout was rated favorably by patients in both visits but more so in visit 2 after the patients had time to work with the plan	*Sample Strategy*: low *Representative sample*: low *Appropriate measures*: low *Nonresponse bias*: unclear *Appropriate analysis*: low *Summary*: no major concerns
Begley [52] Begley [54]	Survey	Diagnosis of epilepsy, aged 18 or older, no major neurological impairments Physicians at participating clinics identified eligible participants	Age: 39.0 (13.05) Female: 26 (60.5%)	• Main: Patients found that MINDSET was easy to use, informative and helpful; raised awareness about epilepsy self-management • Health care providers found MINDSET facilitated identification of self-management challenges, goals, and developing an action plan; felt MINDSET fit into existing workflow	*Sample Strategy*: unclear *Representative sample*: unclear *Appropriate measures*: unclear *Nonresponse bias*: unclear *Appropriate analysis*: low *Summary:* research methods were minimally described; it is unclear if all participants and providers chose to participate and development of measures was not adequately described.
Clark [50] 101 Cross-sectional survey	Survey with closed- and open-ended questions	Currently employed in a position involving services or services related to epilepsy patients or clients; be recognized as making contributions to the understanding of epilepsy in his or her organization or community; be considered a national or international thought leader Study advisors created an initial list of potential participants, and then used snowball sampling to obtain other participants	Age: NR Female: 71%	• Compliance with medical regimen and learning about epilepsy were considered the most important behaviors by about a third of the respondents • 45% of the respondents indicate that finding effective, accessible care was the most significant challenge in the self-management of epilepsy • Barriers faced by clinicians: 37% stated time limitations; 25% stated limited focused training for holistic issues • Challenges as to reasons for medication compliance, and challenges facing clinicians differed between social workers, researches, and clinicians	*Sample Strategy:* unclear *Representative sample:* unclear *Appropriate measures:* unclear *Nonresponse bias:* high *Appropriate analysis:* low *Summary:* research methods minimally described; high non-response bias not adequately addressed.
Fraser [48] 165 Cross-sectional survey (Johnson, [53])	Mailed cross-sectional survey with demographics, relevant epilepsy and behavioral questionnaires, and questions on 13 domains of intervention attributes	Adults 18 and over, epilepsy > 1 year, Recruited by treating neurologist from: epilepsy care centers (*n* = 250) and from a group with self-reported epilepsy from a community support foundation (*n* = 22)	Age: 46.05 (14.02) Female: 92 (56.44%)	• Intervention format: Preference for individual face-to-face sessions (49.69%), followed by face-to-face group (33.33%) and tailored mailed materials (22.01%). Some (39%) preferred multiple-format. Site was unimportant. • Preference for 60-min weekly sessions on weeknight or Saturday afternoons; participants (57%) wanted 8 or fewer sessions, while some (24%) wanted 12 or more sessions • Intervention Leadership: Preference for dyad of leaders—one epilepsy health professional and one PWE.	*Sample Strategy*: unclear *Representative sample*: unclear *Appropriate measures*: low *Nonresponse bias*: unclear *Appropriate analysis:* low *Summary:* minimal description of sampling methods and non-response bias
Johnson [53] Clinicians (*n* = 20); patients (*n* = 165) Cross-sectional survey (Fraser [48])	Patients: Survey had 4 domains (seizure information, general health information, self-management program information, personal background); Clinicians: Survey had 3 domains (epilepsy problem area domains, self-management program, personal background information); mailed self-report survey	Patients: > 18 with chronic epilepsy; Clinicians: medical/allied health providers who treat patients with epilepsy in inpatient/outpatient settings Patients: Epilepsy care medical centers (*n* = 250) and Epilepsy Foundation community support programs (*n* = 22); Clinicians: convenience sample recruited during unit/advisory board meetings; patients: sampling strategy not identified	Age: Clinicians (44.8, SD 11.1); Patients (46, SD 14.0) Female: Clinicians (*n* = 10, 50%); Patients (*n* = 94, 56%)	• Providers indicated importance of personal goal-setting skills and problem-solving approaches, and lower rankings on coping strategies • Program leadership: A different proportion of providers believed that physicians, nurses, and counselors should lead self-management interventions	*Sample Strategy:* low *Representative sample:* unclear *Appropriate measures:* low *Nonresponse bias:* high *Appropriate analysis:* low *Summary:* minimal description of sampling strategy and representativeness; non-response was high with no interpretation presented
Leenen [47] 571 Cross-sectional survey	14-question self-completed or parent-proxy-completed, mailed questionnaire, 12 closed-ended, 2 open-ended questions	Adults with epilepsy or parents/caregivers serving as proxy for their child with epilepsy and/or for people with cognitive deficits associated with their epilepsy. Consecutive series of patients who visited one epilepsy outpatient clinic	Age: 38.3(18.5) Female: 284 (50.7%)	• Self-monitoring: participants engage in self-monitoring behaviors for seizure frequency (16% with e-health tool/digital diary) and/or medication adherence (14% with e-health tool/alarm on phone), change of medication (66%), side effects (43%), use of emergency medications (36%), stress factors (38%) • Possession of hardware: 82% own a computer, 39% own a smartphone. Those 45+ were less likely to use computers or smartphones.	*Sample Strategy:* low *Representative sample:* unclear *Appropriate measures:* low *Nonresponse bias:* unclear *Appropriate analysis:* low *Summary:* reasons for nonresponse were not included
Qualitative studies					
Buelow [40] 25 Qualitative	Semi-structured interviews	Diagnosed with epilepsy for at least 5 years; at least 18 years old; no other major physical or psychological illnesses; was taking at least one antiepileptic medication; had experienced at least one seizure within the last 6 months Purposive and convenience sample of patients identified by physicians, NPs, and the researcher, from a large metropolitan epilepsy center and local epilepsy foundation	Age: 38, range 20-73 Female: NR	• Management techniques (means patients use to manage situations): management of employment and social situations, seizure management, seizure consequences, medications	*Clear Aim*: low *Methods*: low *Appropriate designs*: low *Recruitment*: low *Data collection*: low *Research relationship*: low *Ethical*: unclear *Rigorous analysis*: unclear *Clear findings*: unclear *Valuable research*: unclear *Summary*: minimal description of analytic procedures; no mention of approval from an ethics committee; no description of how the relationship between researcher and participants may have affected the interview process and findings
Laybourne [41] 10 Qualitative (Ridsdale [55])	Individual semi-structured interviews (*n* = 7, 4 in-person, 2 telephone, 1 email) and semi-structured, in-person group interview (1 group, *n* = 3 participants)	Adult PWE with formal diagnosis of epilepsy, prescribed antiepileptic medication, having > 1 seizure in previous 12 months Self-selected study participants	Age: 37 (13.1) Female: 6 (60%)	• Key motivating factor for participation was meeting other PWE, peer support • Other factors: empowerment through knowledge acquisition: being able to bring materials to their partners/caregivers, communicate with medical professionals	*Clear Aim*: low *Methods*: low *Appropriate designs*: low *Recruitment*: low *Data collection*: low *Research relationship*: low *Ethical*: low *Rigorous analysis*: low *Clear findings*: low *Valuable research*: low *Summary*: no major concerns
Ridsdale [22] 20 Nested qualitative study (Ridsdale [55])	Participants were interviewed within 6 months after attending a course from the larger RCT; semi-structured interviews were based on a topic guide (topics generated with service users and piloted prior to use)	Adults aged 16 or over, diagnosis of epilepsy, currently prescribed antiepileptic drugs, have had 2 or more seizures in the previous year, speak/read English Purposively selected from the RCT participants to represent differences in gender, age, ethnicity, and frequency of seizures prior to the RCT	Age: NA Female: 10 (50%)	• Perceived benefits of intervention: Met others with epilepsy; learning method enabled participants to ask questions, share stories, discuss negative feelings, and compare different attitudes/experiences about epilepsy; changing self-management behaviors based on information learned in group • Limitations of intervention: Some participants noted they did not have the language skills or ability to understand what was discussed in the group, some noted memory challenges; some participants reported the course started too early or went too long.	*Clear Aim*: low *Methods*: low *Appropriate designs*: low *Recruitment*: low *Data collection*: low *Research relationship*: low *Ethical*: low *Rigorous analysis*: low *Clear findings*: low *Valuable research*: low *Summary*: no major concerns
Snape [42] 9 health care professionals, 13 PWE, 10 carers Qualitative	Document review, semi-structured individual interviews (face-to-face or telephone; w/health professionals), focus groups (w/PWE using EDs and carers)	Health professionals; PWE/Carers: aged ≥ 16, lived in NW England, provide consent; additionally PWE needed to: have established diagnosis of epilepsy (≥ 1 year), prescribed antiepileptic med, visited an ED in the past 2 years Purposive sampling from intervention groups (PWE/carers), health professionals with specialties/interest in epilepsy; Most professionals were nominated by their discipline’s professional body; PWE and carers identified via user-groups	Age: NA Female: PWE: 6 (46%) Carers: 6 (60%). Health Professionals: NR	• For success in health system, program should be standardized with protocols for health care professionals • Participants valued group format that included carers, • PWE and carers valued written materials (handouts, web access, etc.) to mitigate memory deficits for PWE	*Clear Aim*: low *Methods*: low *Appropriate designs*: low *Recruitment*: low *Data collection*: low *Research relationship*: low *Ethical*: low *Rigorous analysis*: low *Clear findings*: low *Valuable research*: low *Summary*: no major concerns
Walker [43] Pilot: 35; Control: 148 Qualitative - analysis of text from two open-ended questions on social support	Data was derived from the pilot and efficacy WebEase studies. Data collection: within each WebEase module there are text boxes for the person with epilepsy to type in who the support person is and how that person can help them with medication, stress, or sleep behaviors.	Diagnosis of epilepsy; aged 18 or over; English speaking; taking antiepileptic medication for at least 3 months; access to a computer with internet Pilot study participants recruited from two hospital-based clinics; efficacy study (CT) participants recruited via the Internet from epilepsy websites and Listserv	Age: Pilot: 37.5 (12.6); CT: 40.87 (13.32) Female: Pilot: 21 (61.8%); CT: 109 (73.6%)	• 12% of participants indicated no support provider • Spouses and partners, children, friends, siblings, and others were listed as providers • Medication module: Support providers helped with reminders for monitoring medication • Sleep module: support providers helped with strategies to improve sleep, and helped patient reduce stress • Stress module: Support providers provided emotional, appraisal, and instrumental support to the person with epilepsy which helped to reduce stress	*Clear Aim*: low *Methods*: low *Appropriate designs*: low *Recruitment*: low *Data collection*: low *Research relationship*: unclear *Ethical*: low *Rigorous analysis*: low *Clear findings*: low *Valuable research*: low *Summary*: the relationship between the researchers and participants is not well-described
Mixed-methods studies					
Leenen [49] (Leenen [56])	Questionnaires, registration forms, and semi-structured group interviews	Patients: over 18, living at home, diagnosed with epilepsy and used antiepileptic drugs, Relative: invited by patient Recruited from an Academic Center for Epileptology, by press releases in national epilepsy magazines and via social media (Facebook)	Patients Age: 40.5 (13.5) Female: 24 (46.2%) Relatives Age: n/a Female: 21 (56.8%)	• Patients and relatives reported the: program was good; the optimal group size was 10–12 participants; about half the participants stated they expected the program to be more educational; liked the social support from relatives and peer support • Facilitators reported: the optimal group size was 10–12 participants; involvement in intervention was not embedded in regular work duties; wanted motivational interviewing sooner; felt workbook should be simplified and their protocol minimized • Barriers for patients and relatives: technical problems; transportation to intervention location • Barriers for facilitators: could not follow-up with patients to support them, instructions to relatives were unclear	*Rationale*: low *Integrated effectively*: unclear *Integration interpretation*: unclear *Qual quant inconsistencies*: low *Adhere quality*: low *Summary*: minimal description of data integration

Thematic synthesis identified 5 themes that could be applied conceptually to facilitators and barriers. These themes, described in Table 4, included relevance, personalization, intervention components, technology considerations, and clinician interventionist. The presence of themes varied by study as shown in Table 5.

**Table 4:** Themes across studies of self-management of epilepsy

Theme	Definition
Relevance	Relevance of intervention content or topics that facilitate the acquisition of self-management skills in patients with epilepsy
Personalization	Intervention components that account for the individual social, physical, and environmental characteristics of the patient
Intervention components	Components and dosing of the intervention
Technology considerations	Considerations that account for patient’s use, familiarity with, and ownership of technology (e.g., computers, laptops, mobile phones)
Clinician interventionist	Role and preparation of individual who leads the intervention, engages with the patient, and provides self-management education and/or support to the patient

**Table 5:** Presence of themes by study

	Relevance	Personalization	Intervention components	Technology Considerations	Clinician interventionist	Relevance	Personalization	Intervention components	Technology considerations	Clinician interventionist
Study	Facilitators					Barriers				
Atkinson-Clark [51]							X	X		
Begley [52]			X	X				X		
Begley [54]	X		X	X		X			X	
Buelow [40]	X									
Clark [50]	X					X	X			X
Fraser [48]	X		X		X					
Johnson [53]						X				X
Laybourne [41]	X	X	X					X		
Leenen [47]		X		X			X		X	
Leenen [49]			X			X	X	X	X	X
Ridsdale [22]			X			X	X	X		
Snape [42]	X	X	X					X		
Walker [43]		X					X			

### Facilitators

The presence of facilitators of epilepsy self-management interventions at any level (i.e., person, program, site/system) was noted in 11 studies [22, 40–43, 47–50, 52, 54]. Two studies did not include any facilitators [51, 53].

#### Relevance

At the person level, facilitators included desire of the patient with epilepsy to acquire self-management skills [40, 54] and the desire to have content that was highly applicable to living with epilepsy (e.g., through eliciting concerns about self-managing and daily living from the patient or caregiver) (41, 52, 54). At the program level, facilitators included content designed to: enable the patient’s acquisition of skills for living with epilepsy, learn how to apply self-management skills, develop coping strategies for daily life, and communicate with family, caregivers, and health care clinicians about epilepsy [41, 42, 48, 54]. No relevance facilitators were identified at the site/system level.

#### Personalization

At the person level, facilitators included identifying whether the patient owned the necessary technology for the intervention (e.g., computer, mobile telephone) [47], had an identified source of social support (e.g., parents, significant others, friends) [43], and whether the intervention was congruent with the patient’s preference for individual peer-support or group interaction [41, 42]. At the program level, facilitators included developing the intervention and tailoring its components to build on the current self-management practices of the patient [47, 54]. No personalization facilitators were identified at the site/system level.

#### Intervention components

At the person level, facilitators included patient or caregiver receiving written materials (e.g., educational content) to refer to during and after the intervention [42]. At the program level, facilitators included involving family members in the intervention [49], using an empowerment approach to help the patient develop self-management skills [41, 54], and the format of the intervention (e.g., group format that included both the patient and caregiver) [42]. Additional program level factors include the ability to personalize materials to each patient [42, 48, 54], the availability of written materials [41, 42, 52], the ability to interact with a group [22, 49], the provision of peer support [22, 49], and the length and duration of the intervention sessions [22, 49]. At the site/system level, facilitators included developing intervention materials using uniform program standards to ensure program fidelity across intervention sites [42]. One study indicated that the site of the intervention (e.g., medical center) was unimportant, as patients with epilepsy indicated no preference of one site over another [48].

#### Technology considerations

At the program level, facilitators included the high level of usability of intervention components located on the internet, mobile applications, or phones [47, 52, 54]. No technology facilitators were identified at the person or site/system levels.

#### Clinician interventionist

At the program level, facilitators included creating an intervention team consisting of a patient in tandem with an expert health care clinician who could deliver the intervention content [48]. No clinician interventionist facilitators were identified at the person or site/system levels.

### Barriers

The presence of barriers to epilepsy self-management interventions at any level was noted in 11 studies [22, 41–43, 47, 49–54]. Two studies did not include any relevant barriers [40, 48]. We noted that in the studies that addressed barriers, stakeholders included clinicians, social service providers, and researchers [50], and patients and clinicians [53].

#### Relevance

At the program level, barriers included incongruent responses between patients and clinicians about the patient’s problems and needs [53], incongruent opinions by clinicians, researchers, and social service providers on the necessary intervention content [50], and incongruent responses between patients and clinicians on who should lead the intervention and provide epilepsy self-management education and support [53]. Additional barriers at the program level included educational content that was too general or lacking in personalization or tailoring to the patient, his or her disease state and relevant comorbidities [22, 53, 54], and not identifying what the patient views as important in self-management and living with epilepsy [49]. No relevance barriers were identified at the person or site/system levels.

#### Personalization

At the person level, barriers included the patient’s memory and/or cognitive impairments [22, 42, 47, 50, 51, 53], the patient’s disinterest in participating in a self-management intervention [51], not identifying the patient’s preference or desire for self-management support [43], and no current use of the technology by the patient [47]. At the program level, barriers included not accounting for the cognitive limitations of the patient [22]. At the site/system level, barriers included not accounting for the characteristics of the patient population such as the patients’ access to health care [50] or transportation concerns [49].

#### Intervention components

At the program level, barriers included having the patient incur a cost to participate in the intervention [51], not identifying how demographics (e.g., country of origin, burden of disease, socioeconomic status, level of activation) influence the patient’s participation and views of the intervention [51], not identifying the ideal time for follow-up by the clinician after the intervention [49], and not having clear instructions for the role of caregivers participating in or affected by the intervention [49]. Additional program level barriers include not having written materials (e.g., program manuals, handouts, website resources) the patient can refer to during and after the intervention [41, 49], having groups heterogeneously composed of individuals with disparate experiences of living with epilepsy [22], experiencing challenges to scheduling group intervention sessions because of calendar conflicts for participants and clinicians [42], and not identifying the optimal duration and length of the intervention for patients [22, 41, 51]. Barriers at the site/system level included having different levels of attrition at study sites [52] and challenges to using a participatory approach to intervention development and content identification (e.g., lengthy time to complete, need to obtain ethical approval, and efforts to ensure participant engagement) [42]. No intervention component barriers were identified at the person level.

#### Technology considerations

At the person level, barriers included the patient’s lack of knowledge about eHealth tools and functions, having concerns about the privacy of eHealth tools, and varying individual preferences for using technology for epilepsy self-management [47]. At the program level, barriers included difficulty developing eHealth tools with high usability, and a lack of help for users encountering technical difficulties [47, 49, 54]. At the site/system level, barriers included not acknowledging or addressing the inequity of access to eHealth tools within the sample or the person with epilepsy’s concerns about the privacy of eHealth tools [47].

#### Clinician interventionist

At the program level, barriers included not incorporating the duties of the intervention into the clinician interventionist’s normal job duties (i.e., a collateral duty) [49], not adequately preparing the clinician interventionist to deliver the intervention content [49], developing a complex intervention protocol that is difficult to deliver [49], and not identifying the optimal professional role and educational training of the clinician interventionist [50, 53]. At the site/system level, barriers included a lack of opportunity for the clinician interventionist to engage in continuity of care for the person with epilepsy following the conclusion of the intervention [49] and not accounting for the limited time allotted for medical visits [50, 54]. No clinician interventionist barriers were identified at the person level.

### Risk of bias

The tools used to assess risk of bias (ROB) (Table 3, Additional file 3) for the descriptive quantitative, mixed-methods, and qualitative studies did not provide for the calculation of summary scores for individual papers. Among the 7 descriptive quantitative studies, [47, 48, 50–54] ROB was heterogeneous. Patterns that led to judgments of higher ROB included unclear representativeness of the sample (*n* = 6) [47, 48, 50, 51, 53, 54] high (*n* = 2) [50, 53] or unclear (*n* = 4) [48, 51, 53, 54] ROB from non-response, unclear risk of bias in sampling strategy (*n* = 3) [48, 50, 54] and unclear appropriateness of measures (*n* = 2) [50, 54].

## Discussion

This study is the first known systematic review of facilitators and barriers to implementation and adoption of epilepsy self-management interventions. This review aimed to identify facilitators and barriers by examining evidence from diverse studies. We identified five themes: relevance of intervention content, personalization of intervention content and methods, intervention components, technology considerations, and clinician interventionist considerations. Within these themes, facilitators and barriers were identified at the person, program, and system-levels. Our findings add to the literature as recent systematic reviews on self-management interventions for adults with epilepsy [11, 57, 58] have not focused on facilitators and barriers to intervention implementation and adoption (Table 6).

**Table 6:** Highest priority evidence gaps

PICOTS Domain	Evidence Gap
Population	Research is needed with patients who are earlier in their course of illness and studies specifically focused on older adults with epilepsy. Evaluation of interventions and barriers/facilitators to implementation and adoption of self-management interventions with patients and in large health systems is missing.
Interventions	• Self-management interventions are needed that incorporate patient, caregiver, and clinician interventionist input, account for cognitive limitations, incorporate peer support and address other barriers to engagement and adherence. • The role of technology (e.g., smart-phones, web-based support) has not been well studied in patients with epilepsy. • Patients with epilepsy expressed a desire for an intervention team composed of a person with epilepsy and a clinician interventionist to provide self-management education and support. Future research should further examine the composition of this interventionist dyad and identify who the clinician interventionist should be (e.g., registered nurse, advanced practice registered nurse, physician, physician assistant). • Future research should focus on the extent to which these intervention components (e.g., peer support), use of technology, and other identified barriers/facilitators, influence the person with epilepsy’s initial and sustained engagement in an epilepsy self-management program.
Comparators	Active controls, including usual care, are appropriate and should be described carefully.
Outcomes	Future research is needed that specifically addresses the implementation and adoption of epilepsy self-management programs, as there may be additional personal, program, and site/system level barriers that need to be identified and addressed.
Timing	Self-management skills can take time to master and may take longer for patients with cognitive difficulty. Consensus, or research, on the time required to acquire self-management skills and the time required for new skills to potentially improve clinical outcomes is needed.
Setting	Few studies have examined interventions delivered outside of clinical settings. Future research should determine the preferred location for a self-management program for patients with epilepsy and their caregivers.

Our findings underscore a number of key disease-specific considerations for the implementation of epilepsy self-management interventions. Self-management practices that align with an individual’s needs, values, and preferences are considered an important component in delivering patient-centered care [59, 60]. Data in the present review indicates that interventions should be flexible in order to accommodate a person with epilepsy’s preferences about peer support, group interactions, existing self-management strategies, existing self-management social support, and technology preferences. Intervention materials should include written and portable self-management information that is personalized and tailored to the unique situation of each person with epilepsy. Notably, self-management information should be available prior to, during, and after intervention sessions so that individuals with epilepsy, and their caregivers can review and reference self-management content when engaging in self-management behaviors.

Epilepsy providers should incorporate delivery of self-management education and skills into their regular clinical practice to enhance successful delivery of relevant self-management programmatic content. Incorporating the intervention duties into the epilepsy provider’s role and duties will help ensure successful implementation of the intervention in practice and that the interventionist is appropriately trained in the intervention protocol. Overall, our findings indicate that the number of clinical personnel and the time requirements for participation in the intervention are important factors to consider when implementing and adopting interventions. However, no studies in our review addressed the costs related to implementing and adopting self-management programs for epilepsy, nor did any studies address clinician-related costs. Given the personnel and time required for epilepsy self-management programs, new models of care delivery (e.g., telemedicine, mHealth) should be considered as a means to facilitate implementing and adopting epilepsy self-management programs in a cost-effective manner. Virtual care delivery models may be less costly than face-to-face programs and may be more scalable, thus increasing the reach and impact of self-management programs for persons with epilepsy.

One key finding of our study is that no studies directly addressed implementation and adoption issues for epilepsy self-management programs in large health systems. Our included studies addressed factors primarily at the patient level or program level. Important themes that could inform the development, implementation, and/or adoption of future epilepsy self-management interventions included the desire of patients with epilepsy to be involved in the development of intervention content and recognition that cognitive limitations may affect engagement and adherence. Stakeholder engagement is particularly important in developing self-management programs for epilepsy as differences exist among individuals with epilepsy and clinicians in regard to reasons for nonadherence and factors that impact epilepsy self-management [30, 61, 62]. Involving key stakeholders (e.g., patients, caregivers, providers, administrators) may help promote facilitators, and attenuate barriers, to implementing and adopting an epilepsy self-management program. Of note, no studies addressed facilitators and barriers to implementing and adopting epilepsy self-management interventions in relation to cost-effectiveness. Therefore, future research should focus on identifying cost-effective strategies that promote facilitators and reduce barriers to implementation and adoption of epilepsy self-management programs.

Persons with epilepsy expressed a desire for a team composed of an individual with epilepsy and a clinician interventionist to deliver self-management education and support. Thus, future research should further examine the composition of this dyad and identify who the interventionist should be (e.g., registered nurse, advanced practice registered nurse, physician, physician assistant, etc.). One study identified that persons with epilepsy did not have a preference to intervention site such as a medical center or other location. Future research should determine the preferred location for a self-management program for persons with epilepsy and caregivers of persons with epilepsy, with an awareness that access to public transportation may vary widely within a community. Across the studies, persons with epilepsy stated several factors such as access to health care, familiarity and ownership of technology, finances, current self-management support, and transportation that influenced intervention engagement. Future research should focus on the extent to which these factors either individually or in combination influence the person with epilepsy’s initial and sustained engagement in an epilepsy self-management program. We did not identify any study that focused explicitly on the facilitators and barriers to the implementation and adoption of self-management programs for persons with epilepsy for a large health system. Future research is needed that specifically addresses the implementation and adoption of epilepsy self-management programs, as there may be additional personal, program, and site/system level barriers that need to be identified and addressed.

### Limitations and strengths

This is one of the first known systematic reviews to apply rigorous qualitative methods to synthesize data from studies with a wide range of designs (e.g., cross-sectional and longitudinal surveys, discrete-choice experiments, semi-structured interviews, records review, and mixed-methods designs). Using these qualitative evidence synthesis methods, we were able to obtain a more complete understanding of the phenomenon of facilitators and barriers to implementation of epilepsy self-management programs than by comparative methods alone. Yet, several limitations of our study need to be acknowledged. First, while we made every attempt to identify and locate interventions and manuscripts that report on the implementation of epilepsy self-management programs, we may have inadvertently missed relevant articles. Additionally, we limited our search to Organization for Economic Co-operation and Development countries; this search limitation may have inadvertently excluded relevant self-management interventions completed in other countries that could have provided valuable insight into the implementation of epilepsy self-management programs in large health systems. Second, the tools used to assess the risk of bias (ROB) for the studies included in the present review did not allow for the calculation of summary scores. We assessed all studies for ROB. Of the 13 studies, only 1 exhibited a high ROB due to insufficient information about ethical concerns, lack of rigorous analysis of study findings, and no clear value of the research. The remaining studies exhibited either low or unclear ROB. Additionally, we did not contact authors of the studies included in this review to clarify questions pertaining to methods or data collection. Contacting authors to obtain additional information on methods may have influenced our ROB assessment. For the studies in the present review, we addressed the inherent diversity by identifying the respondent (e.g.*,* patient with epilepsy, caregiver, or clinician) and then synthesizing emerging themes within ecological levels.

Our study has a number of strengths. Our review benefited from leveraging input from content experts, being protocol driven, and using rigorous qualitative methods for analyzing barriers and facilitators to implementation and adoption. The use of a theory-based, a priori framework, and a thorough, multiple-investigator review process ensured rigor and validity of our process, findings, and interpretation. By not limiting our inclusion criteria to either quantitative or qualitative designs, we were able to obtain a more comprehensive picture of relevant facilitators and barriers to implementation and adoption of epilepsy self-management programs.

## Conclusion

Findings on facilitators and barriers to implementation underscore key considerations for the design, implementation, and adoption of self-management interventions, including factors of patient personalization, information delivery, use of technology, and intervention personnel. Future research should be designed to address these implementation issues. Future research also should focus on the extent to which intervention components (e.g., peer support), use of technology, and other identified barriers/facilitators influence the person with epilepsy’s initial and sustained engagement in an epilepsy self-management program. In conclusion, our data indicate the importance of incorporating disease-specific considerations when designing, implementing, and adopting an epilepsy self-management program.

## Supplementary information

**Additional file 1.** PRISMA 2019 Checklist

**Additional file 2.** Search Strategy

**Additional file 3.** Risk of Bias (ROB) Assessment

## Data Availability

*Study protocol:* Available at www.crd.york.ac.uk/prospero (PROSPERO: CRD42018098604). *Qualitative data set:* Available from Ms. Gordon (e-mail, adelaide.gordon@va.gov).

## Abbreviations

OECD: Organization for Economic Cooperation and Development
ROB: Risk of bias
