# Supplementary material for: Barriers and facilitators to implementation of epilepsy self-management programs: a systematic review using qualitative evidence synthesis methods
Source: Syst Rev. 2020 Apr 25;9:92. doi: 10.1186/s13643-020-01322-9 (PMC7183113; doi:10.1186/s13643-020-01322-9)
Supplement: Supplementary file 2 — Additional file 2. Search Strategy [file 13643_2020_1322_MOESM2_ESM.docx]

**Additional File 2: Search Strategy**

Reproduced with permission from Luedke MW, Blalock DV, Goldstein KM, et al. Self-management of Epilepsy: A Systematic Review. Ann Intern Med. 2019. <https://annals.org/aim/fullarticle/2737361/self-management-epilepsy-systematic-review?searchresult=1> ©American College of Physicians.

**PubMed: April 13, 2018 and October 31, 2018**

| **Set** | **Terms** | **Results** |
| --- | --- | --- |
| **#1** | "Epilepsy"[Mesh] OR epilepsy[tiab] OR epilepsies[tiab] OR epileptic[tiab] OR epileptics[tiab] OR epilepsia[tiab] | **139,312** |
| **#2** | "Self-Management"[Mesh] OR "Self Care"[Mesh] OR "Self-Control"[Mesh] OR self[tiab] OR selfcare[tiab] OR selfmanagement[tiab] OR selftreatment[tiab] OR selfcontrol[tiab] OR selfhelp[tiab] | **646,690** |
| **#3** | "Patient Compliance"[Mesh] OR "Medication Adherence"[tiab] OR "Medication Compliance"[tiab] OR "Medication nonadherence"[tiab] OR "Medication non adherence"[tiab] OR "Medication Noncompliance"[tiab] OR "Medication non compliance"[tiab] OR "Medication Persistence"[tiab] OR "Health Knowledge, Attitudes, Practice"[Mesh] | **162,567** |
| **#4** | "Behavior Therapy"[Mesh] OR behavior therap*[tiab] OR behaviour therap*[tiab] OR behavioral therap*[tiab] OR behavioural therap*[tiab] OR "anger management"[tiab] OR biofeedback[tiab] OR "bio-feedback"[tiab] OR myobiofeedback[tiab] OR myofeedback[tiab] OR "physiological feedback"[tiab] OR "neuro feedback"[tiab] OR neurofeedback[tiab] OR cognitive therap*[tiab] OR cognition therap*[tiab] OR acceptance therap*[tiab] OR commitment therap*[tiab] OR mindfulness[tiab] OR "MBSR"[tiab] OR "psychologic desensitization"[tiab] OR "psychological desensitization"[tiab] OR "Eye Movement Desensitization and Processing"[tiab] OR EMDR[tiab] OR implosive therap*[tiab] OR exposure therap*[tiab] OR relaxation therap*[tiab] OR "relaxation techniques"[tiab] OR "relaxation technique"[tiab] OR "meditation"[tiab] OR meditate[tiab] OR meditates[tiab] OR "Mind-Body Therapies"[Mesh:NoExp] OR "mind body therapy"[tiab] OR "mind body therapies"[tiab] OR "mind body medicine"[tiab] OR "Breathing Exercises"[Mesh] OR "breathing exercise"[tiab] OR "breathing exercises"[tiab] OR "respiratory muscle training"[tiab] OR "paced respiration"[tiab] OR "Imagery (Psychotherapy)"[Mesh] OR "guided imagery"[tiab] OR "Alexander Technique"[tiab] OR problem-solving therap*[tiab] OR psychodynamic therap*[tiab] OR psychotherap*[tiab] OR "stress reduced"[tiab] OR "stress reducer"[tiab] OR" stress reducers"[tiab] OR "stress reducing"[tiab] OR "stress reduction"[tiab] OR "stress reductions"[tiab] OR "stress reductive"[tiab] | **125,605** |
| **#5** | #1 AND (#2 OR #3 OR #4) AND English[lang] | **3,887** |
| **#6** | #5 NOT (animals[mh] NOT humans[mh]) NOT (("Adolescent"[Mesh] OR "Child"[Mesh] OR "Infant"[Mesh]) NOT "Adult"[Mesh]) | **3,080** |
| **#7** | #6 AND (("randomized controlled trial"[ptyp] OR "controlled clinical trial"[ptyp] OR randomized[tiab] OR randomised[tiab] OR randomization[tiab] OR randomisation[tiab] OR placebo[tiab] OR randomly[tiab] OR trial[tiab] OR groups[tiab] OR "Comparative Study"[ptyp] OR "clinical trial"[pt] OR "clinical trial"[tiab] OR "clinical trials"[tiab] OR "evaluation studies"[ptyp] OR "evaluation studies as topic"[MeSH] OR "evaluation study"[tiab] OR "evaluation studies"[tiab] OR drug therapy[sh] OR "intervention study"[tiab] OR "intervention studies"[tiab] OR "cohort studies"[MeSH] OR cohort[tiab] OR "longitudinal studies"[MeSH] OR longitudinal[tiab] OR longitudinally[tiab] OR prospective[tiab] OR prospectively[tiab] OR "follow up"[tiab] OR "comparative study"[pt] OR "comparative studies"[tiab] OR nonrandom[tiab] OR "non-random"[tiab] OR nonrandomized[tiab] OR "non-randomized"[tiab] OR nonrandomised[tiab] OR "non-randomised"[tiab] OR quasi-experiment*[tiab] OR quasiexperiment*[tiab] OR quasirandom*[tiab] OR quasi-random*[tiab] OR quasi-control*[tiab] OR quasicontrol*[tiab] OR (controlled[tiab] AND (trial[tiab] OR study[tiab])) OR "pre-post"[tiab] OR "posttest"[tiab] OR "post-test"[tiab] OR pretest[tiab] OR pre-test[tiab] OR (before[tiab] AND after[tiab]) OR (before[tiab] AND during[tiab])) NOT (Editorial[ptyp] OR Letter[ptyp] OR Comment[ptyp])) | **1,695** |
| **#8** | #6 AND (("Delivery of Health Care"[Mesh] OR "healthcare delivery"[tiab] OR "health care delivery"[tiab] OR "healthcare system"[tiab] OR "healthcare systems"[tiab] OR "health care system"[tiab] OR "health care systems"[tiab] OR "Health Facilities"[Mesh] OR outpatient[tiab] OR outpatients[tiab] OR clinic[tiab] OR clinics[tiab] OR "primary care"[tiab] OR program[tiab] OR programs[tiab] OR programme[tiab] OR programmes[tiab] OR protocol[tiab] OR protocols[tiab] OR policy[tiab] OR policies[tiab] OR guideline[tiab] OR guidelines[tiab] OR "standards"[Subheading] OR standard[tiab] OR standards[tiab] OR initiative[tiab] OR initiatives[tiab] OR strategy[tiab] OR strategies[tiab] OR "Evidence-Based Practice"[Mesh]) AND ("Program Evaluation"[Mesh] OR "Outcome and Process Assessment (Health Care)"[Mesh] OR "Diffusion of Innovation"[Mesh] OR implement[tiab] OR implements[tiab] OR implementation[tiab] OR implemented[tiab] OR implementing[tiab] OR preimplementation[tiab] OR postimplementation[tiab] OR uptake[tiab] OR adopt*[tiab] OR adapt*[tiab] OR facilitator*[tiab] OR feasible[tiab] OR feasibility[tiab] OR effective[tiab] OR effectiveness[tiab] OR barrier[tiab] OR barriers[tiab] OR benefit[tiab] OR benefits[tiab])) | **568** |
| **#9** | #7 OR #8 | **1,860** |

**Cochrane Central: April 13, 2018**

| **Set** | **Terms** | **Results** |
| --- | --- | --- |
| **#1** | [mh Epilepsy] OR (epilepsy or epilepsies or epileptic or epileptics or epilepsia):ti,ab,kw | **5,832** |
| **#2** | [mh "Self-Management"] OR [mh "Self Care"] OR [mh "Self-Control"] OR (self OR selfcare OR selfmanagement OR selftreatment OR selfcontrol OR selfhelp):ti,ab,kw | **62,762** |
| **#3** | [mh "Patient Compliance"] OR [mh "Health Knowledge, Attitudes, Practice"] OR ("Medication Adherence" OR "Medication Compliance" OR "Medication nonadherence" OR "Medication non adherence" OR "Medication Noncompliance" OR "Medication non compliance" OR "Medication Persistence"):ti,ab,kw | **18,270** |
| **#4** | [mh "Behavior Therapy"] OR [mh^ "Mind-Body Therapies"] OR [mh "Breathing Exercises"] OR [mh "Imagery (Psychotherapy)"] OR ((behavior near/2 therap*) or (behaviour near/2 therap*) or (behavioral near/2 therap*) or (behavioural near/2 therap*) OR "anger management" OR biofeedback OR "bio-feedback" OR myobiofeedback OR myofeedback OR "physiological feedback" OR "neuro feedback" OR neurofeedback OR (cognitive NEAR/2 therap*) OR (cognition NEAR/2 therap*) OR (acceptance NEAR/2 therap*) OR (commitment NEAR/2 therap*) OR mindfulness OR MBSR OR "psychologic desensitization" OR "psychological desensitization" OR "Eye Movement Desensitization and Processing" OR EMDR OR (implosive NEAR/2 therap*) OR (exposure NEAR/2 therap*) OR (relaxation NEAR/2 therap*) OR "relaxation techniques" OR "relaxation technique" OR meditation OR meditate OR meditates OR "mind body therapy" OR "mind body therapies" OR "mind body medicine" OR "breathing exercise" OR "breathing exercises" OR "respiratory muscle training" OR "paced respiration" OR "guided imagery" OR "Alexander Technique" OR ("problem-solving" NEAR/2 therap*) OR (psychodynamic NEAR/2 therap*) OR psychotherap* OR "stress reduced" OR "stress reducer" OR "stress reducers" OR "stress reducing" OR "stress reduction" OR "stress reductions" OR "stress reductive"):ti,ab,kw | **37,550** |
| **#5** | #1 AND (#2 OR #3 OR #4) AND English[lang] | **432** |
| **#6** | #5 NOT (([mh Adolescent] OR [mh Child] OR [mh Infant]) NOT [mh Adult]) | **326** |

**PsycINFO: April 13, 2018**

| **Set** | **Terms** | **Results** |
| --- | --- | --- |
| **S1** | DE "Epilepsy" OR DE "Epileptic Seizures" OR TI ( epilepsy OR epilepsies OR epileptic OR epileptics OR epilepsia) OR AB ( epilepsy OR epilepsies OR epileptic OR epileptics OR epilepsia) | **38,409** |
| **S2** | DE "Self-Management" OR DE "Self-Monitoring" OR DE "Self-Control" OR TI ( self OR selfcare OR selfmanagement OR selftreatment OR selfcontrol OR selfhelp) OR AB ( "self-care*" OR "self-manage*" OR "self-treat*" OR "self-control" OR "self-help" OR "self care" OR "self management" OR "self treatment" OR "self control" OR "self help" OR selfcare OR selfmanagement OR selftreatment OR selfcontrol OR selfhelp) | **160,644** |
| **S3** | DE "Compliance" OR DE "Treatment Compliance" OR DE "Health Attitudes" OR TI ( "Medication Adherence" OR "Medication Compliance" OR "Medication nonadherence" OR "Medication non adherence" OR "Medication Noncompliance" OR "Medication non compliance" OR "Medication Persistence") OR AB ( "Medication Adherence" OR "Medication Compliance" OR "Medication nonadherence" OR "Medication non adherence" OR "Medication Noncompliance" OR "Medication non compliance" OR "Medication Persistence") | **29,260** |
| **S4** | DE "Behavior Therapy" OR DE "Guided Imagery" OR DE "Alternative Medicine" OR DE "Stress and Coping Measures" OR DE "Stress Management" OR DE "Emotional Control" OR DE "Anger Control" OR DE "Relaxation Therapy" OR DE "Progressive Relaxation Therapy" OR DE "Mindfulness" OR AB ( "behavior therap*" OR "behaviour therap*" OR "behavioral therap*" OR "behavioural therap*" OR "anger management" OR biofeedback OR "bio-feedback" OR myobiofeedback OR myofeedback OR "physiological feedback" OR "neuro feedback" OR neurofeedback OR "cognitive therap*" OR "cognition therap*" OR "acceptance therap*" OR "commitment therap*" OR mindfulness OR "MBSR" OR "psychologic desensitization" OR "psychological desensitization" OR "Eye Movement Desensitization and Processing" OR EMDR OR "implosive therap*" OR "exposure therap*" OR "relaxation therap*" OR "relaxation techniques" OR "relaxation technique" OR meditation OR meditate OR meditates OR "mind body therapy" OR "mind body therapies" OR "mind body medicine" OR "breathing exercise" OR "breathing exercises" OR "respiratory muscle training" OR "paced respiration" OR "guided imagery" OR "Alexander Technique" OR "problem-solving therap*" OR "psychodynamic therap*" OR psychotherap* OR "stress reduced" OR "stress reducer" OR "stress reducers" OR "stress reducing" OR "stress reduction" OR "stress reductions" OR "stress reductive" ) OR TI ( "behavior therap*" OR "behaviour therap*" OR "behavioral therap*" OR "behavioural therap*" OR "anger management" OR biofeedback OR "bio-feedback" OR myobiofeedback OR myofeedback OR "physiological feedback" OR "neuro feedback" OR neurofeedback OR "cognitive therap*" OR "cognition therap*" OR "acceptance therap*" OR "commitment therap*" OR mindfulness OR MBSR OR "psychologic desensitization" OR "psychological desensitization" OR "Eye Movement Desensitization and Processing" OR EMDR OR "implosive therap*" OR "exposure therap*" OR "relaxation therap*" OR "relaxation techniques" OR "relaxation technique" OR meditation OR meditate OR meditates OR "mind body therapy" OR "mind body therapies" OR "mind body medicine" OR "breathing exercise" OR "breathing exercises" OR "respiratory muscle training" OR "paced respiration" OR "guided imagery" OR "Alexander Technique" OR "problem-solving therap*" OR "psychodynamic therap*" OR psychotherap* OR "stress reduced" OR "stress reducer" OR "stress reducers" OR "stress reducing" OR "stress reduction" OR "stress reductions" OR "stress reductive" ) | **183,898** |
| **S5** | S1 AND (S2 OR S3 OR S4) Limiters - Publication Type: All Journals; Language: English; Age Groups: Adulthood (18 yrs & older); Population Group: Human; Document Type: Journal Article; Exclude Dissertations | **583** |

**CINAHL: April 13, 2018**

| **Set** | **Terms** | **Results** |
| --- | --- | --- |
| **S1** | (MH "Epilepsy+") OR TI ( epilepsy OR epilepsies OR epileptic OR epileptics OR epilepsia) OR AB ( epilepsy OR epilepsies OR epileptic OR epileptics OR epilepsia) | **18,173** |
| **S2** | (MH "Self Care+") OR TI ( self OR selfcare OR selfmanagement OR selftreatment OR selfcontrol OR selfhelp) OR AB ( "self-care*" OR "self-manage*" OR "self-treat*" OR "self-control" OR "self-help" OR "self care" OR "self management" OR "self treatment" OR "self control" OR "self help" OR selfcare OR selfmanagement OR selftreatment OR selfcontrol OR selfhelp) | **97,606** |
| **S3** | (MH "Patient Compliance+") OR (MH "Attitude to Health+") OR TI ( "Medication Adherence" OR "Medication Compliance" OR "Medication nonadherence" OR "Medication non adherence" OR "Medication Noncompliance" OR "Medication non compliance" OR "Medication Persistence") OR AB ( "Medication Adherence" OR "Medication Compliance" OR "Medication nonadherence" OR "Medication non adherence" OR "Medication Noncompliance" OR "Medication non compliance" OR "Medication Persistence") | **129,591** |
| **S4** | (MH "Behavior Modification+") OR (MH "Guided Imagery") OR (MH "Control (Psychology)+") OR (MH "Biofeedback") OR AB ( "behavior therap*" OR "behaviour therap*" OR "behavioral therap*" OR "behavioural therap*" OR "anger management" OR biofeedback OR "bio-feedback" OR myobiofeedback OR myofeedback OR "physiological feedback" OR "neuro feedback" OR neurofeedback OR "cognitive therap*" OR "cognition therap*" OR "acceptance therap*" OR "commitment therap*" OR mindfulness OR "MBSR" OR "psychologic desensitization" OR "psychological desensitization" OR "Eye Movement Desensitization and Processing" OR EMDR OR "implosive therap*" OR "exposure therap*" OR "relaxation therap*" OR "relaxation techniques" OR "relaxation technique" OR meditation OR meditate OR meditates OR "mind body therapy" OR "mind body therapies" OR "mind body medicine" OR "breathing exercise" OR "breathing exercises" OR "respiratory muscle training" OR "paced respiration" OR "guided imagery" OR "Alexander Technique" OR "problem-solving therap*" OR "psychodynamic therap*" OR psychotherap* OR "stress reduced" OR "stress reducer" OR "stress reducers" OR "stress reducing" OR "stress reduction" OR "stress reductions" OR "stress reductive" ) OR TI ( "behavior therap*" OR "behaviour therap*" OR "behavioral therap*" OR "behavioural therap*" OR "anger management" OR biofeedback OR "bio-feedback" OR myobiofeedback OR myofeedback OR "physiological feedback" OR "neuro feedback" OR neurofeedback OR "cognitive therap*" OR "cognition therap*" OR "acceptance therap*" OR "commitment therap*" OR mindfulness OR MBSR OR "psychologic desensitization" OR "psychological desensitization" OR "Eye Movement Desensitization and Processing" OR EMDR OR "implosive therap*" OR "exposure therap*" OR "relaxation therap*" OR "relaxation techniques" OR "relaxation technique" OR meditation OR meditate OR meditates OR "mind body therapy" OR "mind body therapies" OR "mind body medicine" OR "breathing exercise" OR "breathing exercises" OR "respiratory muscle training" OR "paced respiration" OR "guided imagery" OR "Alexander Technique" OR "problem-solving therap*" OR "psychodynamic therap*" OR psychotherap* OR "stress reduced" OR "stress reducer" OR "stress reducers" OR "stress reducing" OR "stress reduction" OR "stress reductions" OR "stress reductive" ) | **71,641** |
| **S5** | S1 AND (S2 OR S3 OR S4) Limiters - English Language; Age Groups: All Adult; Publication Type: Journal Article | **238** |

**Searches retrieved 2,996 records before duplicates were removed.**
