# Supplementary material for: Barriers and facilitators to implementation of epilepsy self-management programs: a systematic review using qualitative evidence synthesis methods
Source: Syst Rev. 2020 Apr 25;9:92. doi: 10.1186/s13643-020-01322-9 (PMC7183113; doi:10.1186/s13643-020-01322-9)
Supplement: Supplementary file 3 — Additional file 3. Risk of Bias (ROB) Assessment [file 13643_2020_1322_MOESM3_ESM.docx]

**Additional File 3: Risk of Bias (ROB) Assessment**

**Figure Legend**

**AF 1. Risk of Bias Ratings for the Included Qualitative Studies**

**AF 2. Risk of Bias Ratings for the Included Descriptive Quantitative Studies**

**AF 3. Risk of Bias Ratings for the Included Mixed-Methods Study**

**Summary ROB ratings are defined as follows:**

- Low ROB: Bias, if present, is unlikely to alter the results seriously.
- Unclear ROB: A risk of bias that raises some doubts about the results.
- High ROB: Bias may alter the results seriously.

**Qualitative studies**

We used the 10-item Critical Appraisal Skills Programme (CASP) for Qualitative Research Studies [1] for the included qualitative studies (n=5). Each item is rated “Yes,” “No,” or “Can’t tell”; there is no summary rating. The overall ROB in the 5 qualitative studies was low (**AF 1)** [2-6]. However, we identified several concerning flaws in 1 study [2] including insufficient information provided regarding ethical considerations or consideration of the relationship between the researcher and participants, a lack of rigorous analysis of study findings, and no description of the clear value of the research. We also identified 1 study that had unclear ROB regarding the relationship of the researcher to the participants [6].

**
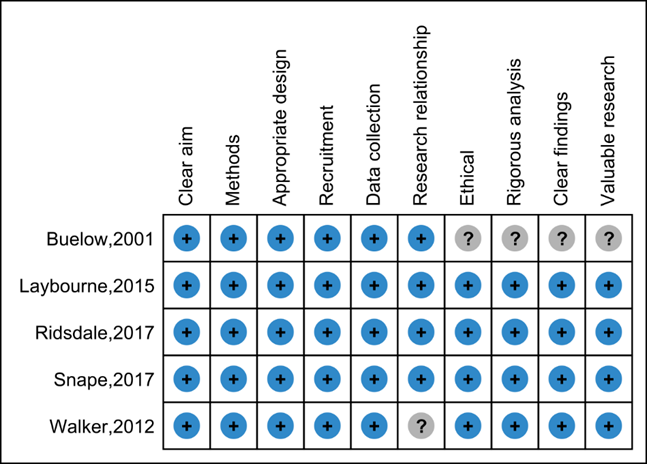
**

**AF 1. Blue/positive indicates items that were judged low ROB. Gray/question mark indicates items that were judged unclear ROB.**

**Quantitative descriptive studies**

For the quantitative descriptive studies, we used the 5 items in the Mixed Methods Appraisal Tool (MMAT) [7]. These criteria address the sampling strategy, the sample representativeness, measurements, risk of nonresponse bias, and appropriateness of the statistical analysis. The MMAT rates each item “Yes,” “No,” or “Can’t tell”; there is no summary rating. Among the 7 descriptive quantitative studies [8-14], ROB was heterogeneous (**AF 2**). Patterns that led to judgments of higher ROB included unclear representativeness of the sample (n=6) [8, 10-14], high (n=2) [10, 12], or unclear (n=4) [9, 11, 13, 14]. ROB from non-response, unclear risk of bias in sampling strategy (n=3) [10, 11, 14] and unclear appropriateness of measures (n=2) [10, 14].


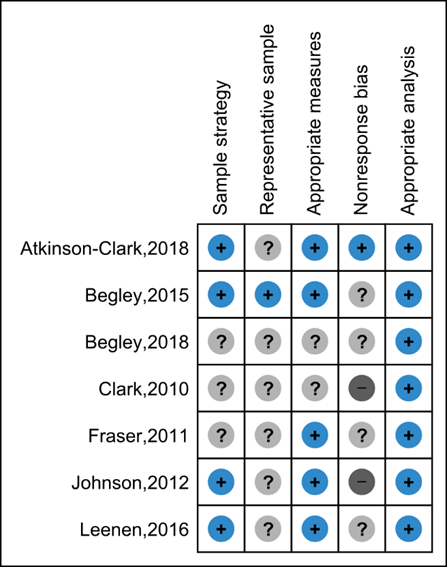


**AF 2. Blue/positive indicates items that were judged low ROB. Light gray/question mark indicates items that were judged unclear ROB. Dark gray/negative indicates items that were judged high ROB.**

**Mixed-methods study**

We used the 5-item MMAT specific to mixed-methods studies [7] for the mixed-methods study (n=1). These criteria address the rationale for using mixed methods, the integration of the study components, the interpretation of the study components, discussion of divergences or inconsistencies between the quantitative and qualitative data, and how each component of the study adheres to the quality criteria of each of the quantitative and qualitative methods. Similar to the quantitative descriptive studies, the MMAT for mixed-methods studies rates each item “Yes,” “No,” or “Can’t tell”; there is no summary rating. The ROB of the mixed-methods study was somewhat unclear, as it had no integration of its qualitative and quantitative findings (**AF 3**) [15].


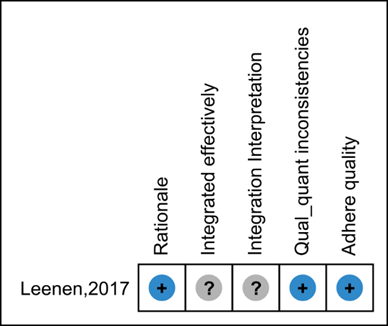


**AF 3. Blue/positive indicates items that were judged low ROB. Gray/question mark indicates items that were judged unclear ROB.**

**References in Additional File 3**

1. Anonymous. Critical Appraisal Skills Programme (CASP). CASP Qualitative Checklist. Available at: <http://www.casp-uk.net/casp-tools-checklists>. Accessed May 17, 2018.

2. Buelow JM. Epilepsy management issues and techniques. J Neurosci Nurs. 2001;33(5):260-9. PMID: 107067167. Language: English. Entry Date: 20011109. Revision Date: 20150818. Publication Type: Journal Article.

3. Laybourne AH, Morgan M, Watkins SH, Lawton R, Ridsdale L, Goldstein LH. Self-management for people with poorly controlled epilepsy: Participants' views of the UK Self-Management in epILEpsy (SMILE) program. Epilepsy Behav. 2015;52(Pt A):159-64. doi: 10.1016/j.yebeh.2015.08.023. PMID: 26426353.

4. Ridsdale L, Philpott SJ, Krooupa AM, Morgan M. People with epilepsy obtain added value from education in groups: results of a qualitative study. Eur J Neurol. 2017;24(4):609-16. doi: 10.1111/ene.13253. PMID: 28181344.

5. Snape DA, Morgan M, Ridsdale L, Goodacre S, Marson AG, Noble AJ. Developing and assessing the acceptability of an epilepsy first aid training intervention for patients who visit UK emergency departments: A multi-method study of patients and professionals. Epilepsy Behav. 2017;68:177-85. doi: 10.1016/j.yebeh.2017.01.006. PMID: 28213317.

6. Walker ER, Bamps Y, Burdett A, Rothkopf J, Diiorio C. Social support for self-management behaviors among people with epilepsy: a content analysis of the WebEase program. Epilepsy Behav. 2012;23(3):285-90. doi: 10.1016/j.yebeh.2012.01.006. PMID: 22364762.

7. Hong QN, Pluye P, Fàbregues S, Bartlett G, Boardman F, Cargo M, Dagenais P, Gagnon M-P, Griffiths F, Nicolau B, O’Cathain A, Rousseau M-C, Vedel I. Mixed Methods Appraisal Tool (MMAT), version 2018. Registration of Copyright (#1148552), Canadian Intellectual Property Office, Industry Canada. Available at: <http://mixedmethodsappraisaltoolpublic.pbworks.com/w/page/127425845/Download%20the%20MMAT>. Accessed October 11, 2018.

8. Atkinson-Clark E, Charokopou M, Van Osselaer N, Hiligsmann M. A discrete-choice experiment to elicit preferences of patients with epilepsy for self-management programs. Epilepsy Behav. 2018;79:58-67. doi: 10.1016/j.yebeh.2017.11.015. PMID: 29248866.

9. Begley C, Shegog R, Harding A, Goldsmith C, Hope O, Newmark M. Longitudinal feasibility of MINDSET: a clinic decision aid for epilepsy self-management. Epilepsy Behav. 2015;44:143-50. doi: 10.1016/j.yebeh.2014.12.031. PMID: 25705825.

10. Clark NM, Stoll S, Youatt EJ, Sweetman M, Derry R, Gorelick A. Fostering epilepsy self management: The perspectives of professionals. Epilepsy Behav. 2010;19(3):255-63. doi: 10.1016/j.yebeh.2010.08.033. PMID: 2010-26671-010.

11. Fraser RT, Johnson EK, Miller JW, Temkin N, Barber J, Caylor L, Ciechanowski P, Chaytor N. Managing epilepsy well: Self-management needs assessment. Epilepsy Behav. 2011;20(2):291-8. doi: 10.1016/j.yebeh.2010.10.010.

12. Johnson EK, Fraser RT, Miller JW, Temkin N, Barber J, Caylor L, Ciechanowski P, Chaytor N. A comparison of epilepsy self-management needs: provider and patient perspectives. Epilepsy Behav. 2012;25(2):150-5. doi: 10.1016/j.yebeh.2012.07.020. PMID: 23032121.

13. Leenen LAM, Wijnen BFM, de Kinderen RJA, van Heugten CM, Evers SMAA, Majoie MHJM. Are people with epilepsy using eHealth-tools? Epilepsy Behav. 2016;64(Part A):268-72. doi: 10.1016/j.yebeh.2016.08.007. PMID: 2016-57261-046.

14. Begley C, Chong J, Shegog R, Sepulveda R, Halavacs N, Addy R, Martin K, Labiner D. MINDSET: Clinical feasibility of utilizing the revised epilepsy self-management tool for Spanish speaking patients. Epilepsy Behav. 2018;88:218-26. doi: 10.1016/j.yebeh.2018.09.021. PMID: 30300871.

15. Leenen LAM, Wijnen BFM, van Haastregt JCM, de Kinderen RJA, Evers S, Majoie M, van Heugten CM. Process evaluation of a multi-component self-management intervention for adults with epilepsy (ZMILE study). Epilepsy Behav. 2017;73:64-70. doi: 10.1016/j.yebeh.2017.05.023. PMID: 28623752.
